# Retracted: Correlations of Ezrin Expression with Pathological Characteristics and Prognosis of Osteosarcoma: A Meta-Analysis

**DOI:** 10.1155/2022/9860781

**Published:** 2022-10-24

**Authors:** The Scientific World Journal

*The Scientific World Journal* has retracted the article titled “Correlations of Ezrin Expression with Pathological Characteristics and Prognosis of Osteosarcoma: A Meta-Analysis” [1]. This article is one of a series of very similar meta-analyses written by different authors that were published in 2014 and 2015, characterized by searching the complementary and alternative medicine database CISCOM despite the topic not being about CAM [2]. In addition, it was identified that the original files submitted by the authors were edited by MedChina, a company previously alleged to be involved in the sale of articles [2].

The authors responded to explain that MedChina provided only language editing services on this article and that some mistakes were made due to this being their first systematic review publication. The authors agree to the retraction and the notice.

## References

[1] Zhao D.-H., Zhu J., Wang W.-B. (2014). Correlations of Ezrin Expression with Pathological Characteristics and Prognosis of Osteosarcoma: A Meta-Analysis. *The Scientific World Journal*.

[2] Seife C. (2014). For sale: “Your name here” in a prestigious science journal. *Scientific American*.

